# Amifostine ameliorates bleomycin-induced murine pulmonary fibrosis via NAD^+^/SIRT1/AMPK pathway-mediated effects on mitochondrial function and cellular metabolism

**DOI:** 10.1186/s40001-023-01623-4

**Published:** 2024-01-20

**Authors:** Feng Guo, Feng Xu, Shujuan Li, Yun Zhang, Dan Lv, Lin Zheng, Yongxiong Gan, Miao Zhou, Keyu Zhao, Shuling Xu, Bin Wu, Zaichun Deng, Panfeng Fu

**Affiliations:** 1 Department of Biochemistry, Health Science Center, Ningbo University, Ningbo, 315041 China; 2 Central Laboratory of the Medical Research Center, The First Affiliated Hospital of Ningbo University, Ningbo, China; 3 Department of Respiratory and Critical Care Medicine, The First Affiliated Hospital of Ningbo University, Ningbo University, Ningbo, 315041 China; 4 Department of Laboratory Medicine, The First Affiliated Hospital of Ningbo University, Ningbo, China; 5 Department of Emergency Medicine, The First Affiliated Hospital of Ningbo University, Ningbo, China; 6 Department of Dermatology, The First Affiliated Hospital of Ningbo University, Ningbo, China; 7 Department of Pulmonary and Critical Care Medicine, South China Hospital Affiliated to Shenzhen University, Shenzhen, China

**Keywords:** Amifostine, IPF, TGF-β1, Mitochondria, Metabolism, ROS, SIRT1, NAD^+^

## Abstract

**Background:**

Idiopathic pulmonary fibrosis (IPF) is a devastating chronic lung disease characterized by irreversible scarring of the lung parenchyma. Despite various interventions aimed at mitigating several different molecular aspects of the disease, only two drugs with limited clinical efficacy have so far been approved for IPF therapy.

**Objective:**

We investigated the therapeutic efficacy of amifostine, a detoxifying drug clinically used for radiation-caused cytotoxicity, in bleomycin-induced murine pulmonary fibrosis.

**Methods:**

C57BL6/J mice were intratracheally instilled with 3 U/kg of bleomycin. Three doses of amifostine (WR-2721, 200 mg/kg) were administered intraperitoneally on days 1, 3, and 5 after the bleomycin challenge. Bronchoalveolar lavage fluid (BALF) was collected on day 7 and day 21 for the assessment of lung inflammation, metabolites, and fibrotic injury. Human fibroblasts were treated in vitro with transforming growth factor beta 1 (TGF-β1), followed by amifostine (WR-1065, 1–4 µg/mL) treatment. The effects of TGF-β1 and amifostine on the mitochondrial production of reactive oxygen species (ROS) were assessed by live cell imaging of MitoSOX. Cellular metabolism was assessed by the extracellular acidification rate (ECAR), the oxygen consumption rate (OCR), and the concentrations of various energy-related metabolites as measured by mass spectrum (MS). Western blot analysis was performed to investigate the effect of amifostine on sirtuin 1 (SIRT1) and adenosine monophosphate activated kinase (AMPK).

**Results:**

Three doses of amifostine significantly attenuated lung inflammation and pulmonary fibrosis. Pretreatment and post-treatment of human fibroblast cells with amifostine blocked TGF-β1-induced mitochondrial ROS production and mitochondrial dysfunction in human fibroblast cells. Further, treatment of fibroblasts with TGF-β1 shifted energy metabolism away from mitochondrial oxidative phosphorylation (OXPHOS) and towards glycolysis, as observed by an altered metabolite profile including a decreased ratio of NAD + /NADH and increased lactate concentration. Treatment with amifostine significantly restored energy metabolism and activated SIRT1, which in turn activated AMPK. The activation of AMPK was required to mediate the effects of amifostine on mitochondrial homeostasis and pulmonary fibrosis. This study provides evidence that repurposing of the clinically used drug amifostine may have therapeutic applications for IPF treatment.

**Conclusion:**

Amifostine inhibits bleomycin-induced pulmonary fibrosis by restoring mitochondrial function and cellular metabolism.

**Supplementary Information:**

The online version contains supplementary material available at 10.1186/s40001-023-01623-4.

## Introduction

Idiopathic pulmonary fibrosis (IPF) is a progressive and devastating disease characterized by aberrant fibroblast proliferation, excessive accumulation of collagen, and the destruction of lung structure. The etiology of this disease is not completely understood, thus presenting challenge for its treatment. Oxidative stress represents a key molecular mechanism in the etiology of IPF. Reactive oxygen species (ROS) are generated by enzymatic systems including NADPH oxidase (NOX), xanthine oxidase, nitric oxide synthase (NOS), and the mitochondrial electron transport chain. ROS generated in the mitochondria appear to be important in mediating pulmonary fibrosis [1, 2]. Mitochondrial ROS directly cause mitochondrial DNA release and protein oxidation, as well as being coupled to mitochondrial metabolic changes [3]. These are recognized as distinctive features of IPF lungs [4–6]. Transforming growth factor beta (TGF-β) is the most potent profibrogenic cytokine in nearly all fibrotic diseases, and has been shown to increase mitochondrial ROS and induce metabolomic reprogramming [7–9]. ROS and metabolomic reprogramming may synergistically promote fibroblast proliferation, collagen production, and induction of the epithelial-mesenchymal transition (EMT).

Metabolic changes are increasingly recognized as being a significant driver of fibrosis in many different organs. Fibrotic foci of lung tissue in IPF have altered metabolic activity [10]. Several aspects of metabolic change may contribute to the pathogenesis of IPF, including an imbalance of glycolysis and oxidative phosphorylation (OXPHOS), mitochondrial dysfunction, alterations in glutaminolysis, and decreased lipid acid oxidation [11]. Interventions that target mitochondrial ROS and metabolic perturbations have, therefore, been widely investigated. For example, the combined use of senolytics and antioxidants can ameliorate fibrosis by cleaning senescent cells [12]. Rapamycin, an inhibitor of the mTOR pathway, shows anti-fibrotic therapeutic effects in combination with pirfenidone [13]. Sirtuins (SIRTs) are a family of class III histone deacetylases, with some members having well documented roles in multiple organ fibrosis. SIRT1 has been shown to regulate canonical TGF-β signaling, thereby controlling fibroblast activation and tissue fibrosis [14]. SIRT3 deficiency promotes lung fibrosis by increasing mitochondrial DNA damage in alveolar epithelial cells leading to apoptosis [15]. Whole-body overexpression of SIRT3 protects mice from asbestos-induced pulmonary fibrosis by mitigating lung mtDNA damage [16]. In addition, the synthetic compound C75, a fatty acid synthase inhibitor, can maintain renal cell viability and reduce fibrosis in rodent models [17]. Peroxisome proliferator-activated receptors (PPARs) are a set of transcription factors that regulate lipid metabolism and inflammation. Agonists targeting PPARs have been shown to attenuate lung fibrosis [18]. Despite advances in anti-fibrosis drug develoment only two drugs, pirfenidone and nintedanib, are approved for IPF patients having a prognosis of approximately 2–4 years median survival after diagnosis [19]. Both have limited efficacy and therefore more effective pharmacological approaches are urgently needed.

Amifostine is a thiophosphate prodrug approved by the American Food and Drug Administration (FDA) for the prevention of toxicities associated with cisplatin therapy and therapeutic radiation [20]. It functions as an oxygen and ROS scavenger. We previously showed that amifostine protected mice from lipopolysaccharide (LPS)-induced acute lung injury (ALI) and ventilator-induced lung injury (VILI) [21, 22]. In the present study, we reasoned that amifostine may inhibit fibrosis in a bleomycin-induced murine pulmonary fibrosis model by reducing oxidative stress and protecting mitochondrial dysfunction. We found that amifostine significantly attenuated bleomycin-induced pulmonary fibrosis and improved the survival rate. We also demonstrated that amifostine reduced the production of TGF-β1-induced fibroblast mitochondrial ROS, restored mitochondrial function, and rescued adenosine mono-phosphate activated kinase (AMPK) in a SIRT1-dependent manner. Taken together, these findings suggest that amifostine may have a therapeutic role for IPF by modulating mitochondrial metabolism and function.

## Materials and methods

### Animals

Male C57BL6/J mice aged 8 weeks were purchased from the Zhejiang Weitong Lihua Experimental Animal Company, China, and housed in pathogen-free conditions with food and water. All animal care and experimental procedures were approved by the Ningbo University Animal Care and Use Committee.

### Bleomycin treatment

Mice were anesthetized with an intraperitoneal injection of ketamine (75 mg/kg) and bleomycin sulphate (Hanhui Pharmaceutical Ltd. Co., China) dissolved in 0.9% saline was then intratracheally instilled (3 U/kg dose in 30 µL). Three doses of amifostine (WR-2721, 200 mg/kg) or sterile PBS were given intraperitoneally on day 1, 3 and 5. The body weight of each mouse was measured every second day from day 1 until day 14. In parallel, mice were intratracheally instilled with 20 µL PBS or with 5 U/kg bleomycin for the survival experiments. The survival of mice was monitored until day 14.

### Analysis of bronchoalveolar lavage analysis

Mice were sacrificed on days 14 and 21 and bronchoalveolar lavage fluid (BALF) was taken. Samples were centrifuged at 300 × g, the supernatant was collected and further centrifuged at 12,000 × g for assay with a Pierce™ BAC protein assay kit (Thermo Fisher Scientific, 23225). BAL cytokine levels were measured by ProcartaPlex™ Multiplex Immunoassay (Invitrogen, EPX360-26092-901) with a Luminex™ 100 instrument according to the manufacture’s instructions. The pellets from the first centrifuge were resuspended in PBS and the total and differential cell counts analyzed as previously described [21].

### Histology analysis

Following collection of BALF, the right lung lobes were collected and stored at – 80 ℃ for Western blot. The left lobe was collected and preserved in 10% neutral buffered formalin for at least 24 h. The lung tissue was then sectioned (5 µm) and stained with hematoxylin and eosin (H&E). Lung injury was scored according to the American Thoracic Society workshop report [23].

### Mitochondrial ROS detection in live cells

Live cell mitochondrial ROS was assessed using commercial MitoSOX red dye (Invitrogen, M36008) according to the kit instructions. Briefly, primary human fibroblast cells (NHLP, from Lonza, can# CC-2512) cultured in RPMI 1640 medium were treated with bleomycin or amifostine (WR-1065, 4 µg/mL) and MitoSOX red was added to the medium to reach 5 µM. Cells were incubated for 10 min at 37 °C, then washed three times with warm PBS buffer. Mitochondrial ROS were visualized by confocal microscope at an excitation wavelength of 510 nm.

### Intracellular ATP measurement

NHLP cells grown in 100 culture dishes were pre-treated with TGF-β1 for one hour followed by amifostine (1–4 µg/mL) treatment for another hour. Cells were then detached using trypsin and washed in ice-cold PBS. Cell pellets were obtained by centrifugation at 1,000 g for 10 min. The cells were then resuspended in 0.5 mL ice-cold PBS and ATP was measured using an ATP Bioluminescence Assay Kit HSII (Millipore) according to the manufacturer’s instructions. Data are presented as nmol ATP/mg protein.

### Metabolic analysis with seahorse XF96 analyzer

Human fibroblast cells were plated at a density of 150,000 cells/well in 50 µL of balanced XF DMEM assay medium (pH 7.4) supplemented with 10 mM XF glucose, 2 mM XF glutamine and 1 mM XF pyruvate. Cells were pre-treated with saline or TGF-β1 for 30 min, followed by saline or amifostine (4 µg/mL) treatment for another 30 min. After the above treatments, an additional volume of 130 µL assay medium was added and the cells were incubated for another 20 min. XF assays were performed using serial injections of 1.5 µM Oligomycin, 1 µM FCCP, and a combination of 1.25 µM Rotenone and 2.5 µM Antimycin A. Measurements were carried out using 12 cycles of 3 min, with 2 min of mixing in between measurements.

### Metabolites measurement by LC–MS

NHLP cells grown in 100 culture dishes were pre-treated with TGF-β1 for one hour followed by amifostine (1–4 µg/mL) treatment for another hour. Cells were then collected in 0.5 mL of ice-cold extraction solvent (MeOH:PBS:Water, 8:1:1, v/v/v). Cells were then centrifuged at 12,000 × g for 15 min at 4 °C. The supernatant was transferred into a new tube and subjected to vacuum drying at 30 °C for 1 h. After samples drying, add 500 µL of H_2_O/PBS/ACN (2:2:6) solution into the pellet to solve the sample. Transfer 50 µL of prepared sample to run LC–MS for quality control. The rest sample was used for measurement of metabolites.

### Western blotting

Cells were lysed in RIPA buffer supplemented with protease and phosphatase inhibitors (Thermo Fisher Scientific, 78446). Electrophoresis and immunoblotting were performed as previously described [22]. The following primary antibodies purchased from Cell Signaling Technology were used for the Western blotting analyses: anti-α-SMA (#19245), anti-fibronectin (#26836), anti-collagen 1A1 (#72026), anti-phospho-AMPK (#2535), anti-AMPK (#2532) and anti-SIRT1 (#8469). Anti-tubulin antibody was obtained from Sigma–Aldrich (#t6199). Following incubation with the corresponding primary antibodies, membranes were incubated with horseradish peroxidase-conjugated secondary antibody for one hour and immune reactive bands were then visualized by enhanced chemiluminescence (ECL) detection.

### SiRNA transfection

Human fibroblast cells in 6-well plates were cultured to 90% confluence and then transfected with non-specific RNA (nsRNA), siAMPK, or siSIRT1 RNA mixed with Lipofectamine 2000 (Invitrogen, 11668030) at a dose of 100 nmol/L for 24 h. After transfection, fresh medium was added and the cells were cultured for another 24 h before use. Protein levels were assessed by Western blot.

### SIRT1 activity assay

Following treatment with bleomycin and/or amifostine, human fibroblast cells cultured in D100 dishes were collected in ice-cold non-denaturing cell lysate buffer (20 mM Tris–HCl [pH 7.5], 250 mM NaCl, 1 mM EDTA, 1% Triton X-100, 1 mM DTT). After brief sonication, the samples were centrifuged at 12,000 g for 10 min at 4 °C. The supernatant was collected for SIRT1 activity measurement according to the abcam SIRT1 activity assay kit (ab156065). Briefly, 20 µL of cell lysate or SIRT1-purified enzyme was added to a 96-well plate, 10 µL of SIRT1 substrate solution was added to each well and mixed. The plate was then incubated at 37 °C for 30 min before adding 5 µL of developing solution and incubating at 37 °C for a further 10 min. The fluorescence was then measured by fluorometer for 20 min at an excitation wavelength of 340–380 nm and an emission wavelength of 430–460 nm. The activity was determined by the ratio of fluorescence intensity to time.

### Statistical analysis

Data are expressed as mean ± SEM unless otherwise specified. One-way ANOVA with post-hoc Bonferroni test was used for multiple group comparisons. The log-rank test was used to compare differences in survival between two groups. Multiple comparisons were performed for metabolite analysis. P-values were adjusted by Tukey’s test. A two-sided p-value < 0.05 was considered statistically significant. All analyses were performed with GraphPad Prism version 9.0 (GraphPad Software).

## Results

### Amifositne protects mice against bleomycin-induced lung fibrosis

**Fig. 1:**
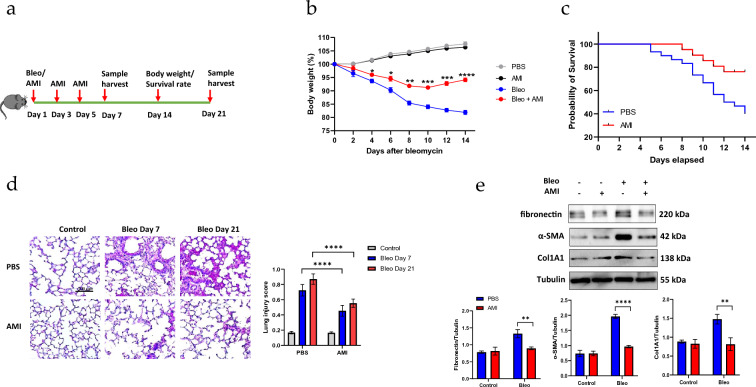
Amifostine attenuates bleomycin-induced IPF.** a** Experimental design. C57BL mice were intratracheally injected with bleomycin on day 1. Three doses of intraperitoneal amifostine were administered on day 1, 3 and 5. Animals were sacrificed on day 7 or day 21, BAL and lung tissue samples were collected. **b** Animal body weights were measured every second day until day 14, *n* = 10, *p < 0.05, **p < 0.01, ***p < 0.001, ****p < 0.0001. **c** Animal survival experiment was performed with high dose of bleomycin (5 U/kg). The survival rate was examined over 14 days *n *= 20, *p* < 0.001. **d** Histological H&E staining of lung tissues from control mice and from bleomycin-treated mice at days 7 and 21. Lung injury was evaluated as described in the Materials and Methods. *n* = 10, ****p < 0.0001. **e** The lung tissue fibrosis markers fibronectin, α-SMA and collagen1A1 were detected by Western blot. The results shown are cropped from original blots

We first examined whether intraperitoneal injection of mice with amifostine could prevent intratracheal bleomycin-induced lung fibrosis. C57BL6 mice were intratracheally instilled with sterile PBS or bleomycin. Bronchial alveolar lavage (BAL) fluid was collected on days 7 and 21 after bleomycin challenge to analyze the initial inflammation response and the later fibrotic response (Fig. 1a). Compared with PBS control mice, bleomycin-treated mice had a lower body weight from day 2 and this continued to day 14 after bleomycin treatment. Post-treatment with amifostine significantly attenuated the body weight loss induced by bleomycin, with the body weight loss recovering by day 10 (Fig. 1b). Amifostine by itself had no effect on body weight compared with the saline control. Furthermore, the survival of PBS-treated control mice at day 14 was only 44.75% after bleomycin challenge, which was significantly lower than that of amifostine-treated mice (84.37%, *p* = 0.0199) (Fig. 1c). The hazard ratio between the PBS and amifostine-treated mice was 2.828, with a 95% CI of 1.220–6.558. Histological analysis showed that bleomycine caused profound lung inflammation on day 7, but this was attenuated by amifostine (Fig. 1d). The inflammatory response was still obvious on day 21. Meanwhile, alveolar septum was significantly thickened with obvious fibrosis developed in the interstitial. In contrast, amifostine-treated mice presented less inflammation and fibrotic deposit in the interstitial tissue. We further investigated lung fibrosis by examining the expression of fibrosis makers with Western blot. This showed that bleomycin induced the expression of fibrosis marker proteins such as collagen, fibronectin, and α-SMA (Fig. 1e). However, treatment with amifostine significantly attenuated the expression of these markers (Additional file 1).

**Fig. 2:**
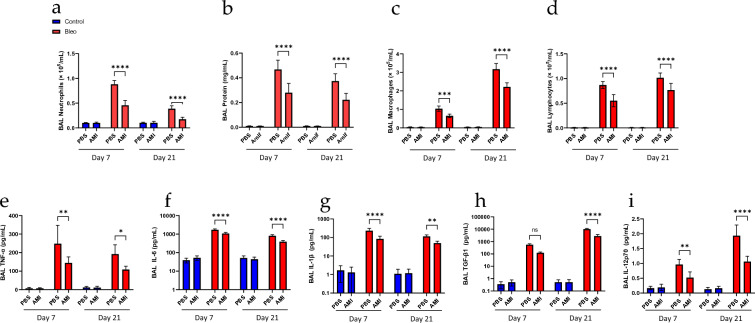
Amifostine attenuates bleomycin-induced pulmonary immunological responses. Lung injury and immunological responses were assessed by the analysis of BAL samples collected on day 7 and day 21. **a, c, d** Neutrophils, macrophages and lymphocytes were quantified. **b** BAL protein was also measured**. e–i** Proinflammatory and profibrotic cytokines were analyzed. *n* = 10, *p < 0.05, **p < 0.01, ***p < 0.001, ****p < 0.0001

We next assessed BAL fluid for the inflammatory responses. This analysis revealed distinct inflammatory and profibrotic responses at day 7 and day 21. As shown in Fig. 2, inflammatory responses including BAL protein content, neutrophils infiltration and proinflammatory cytokine (IL-6, IL-1β, TNF-α) release were significantly upregulated on day 7 and persisted to day 21. However, the levels on day 21 were lower than on day 7, indicating the acute inflammatory responses on day 7 preceded the fibrotic phase on day 21. In contrast, profibrotic responses were upregulated, including the accumulation of BAL macrophages and lymphocytes, and increased TGF-β and IL-12p70 levels, thereby indicating development of the fibrotic phase. Of note, amifostine treatment significantly suppressed both the inflammatory and fibrotic responses. Collectively, these in vivo results clearly demonstrate the therapeutic effects of amifostine on bleomycin-induced pulmonary fibrosis in a mouse model.

### Amifostine reverses mitochondrial dysfunction induced by TGF-β1

**Fig. 3:**
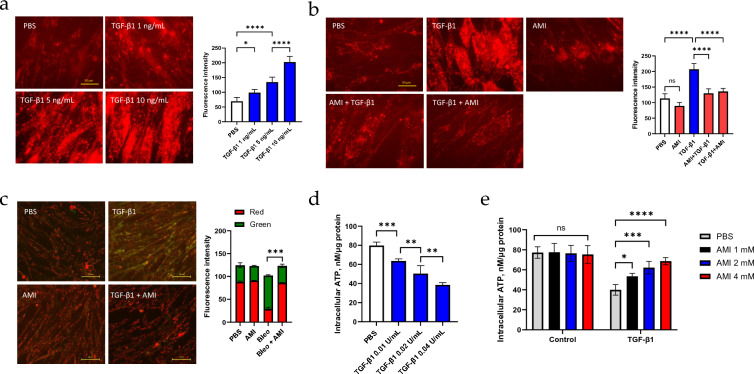
Amifostine improves mitochondrial oxidative stress and mitochondrial function. **a** Cells were treated with various doses of TGF-β1 and then mitochondrial oxidative stress was measured by MitoSOX. At least 10 slides were used for quantification. *p < 0.05, ****p < 0.0001. **b** Mitochondrial ROS quantification. ****p < 0.0001, *n* = 10–15. **c** Mitochondrial function was assessed by measuring the mitochondrial membrane potential. Images were obtained separately in the red and green channels and are presented as a merged image. Red color represents normal mitochondrial potential, while green color represents compromised mitochondrial potential. **d** and **e** Intracellular ATP levels. *p < 0.05, **p < 0.01, ***p < 0.001, ****p < 0.0001

Amifostine has long been considered as a ROS scavenger. Here, we investigated its effect on mitochondrial ROS, since mitochondrial oxidative stress is known to play a vital role in the development of IPF. TGF-β1 is the key cytokine that mediate bleomycin-induced fibrosis and is widely used to induce a fibrotic phenotype in various in vitro models [24, 25]. We, therefore, used TGF-β1 for all of the current in vitro experiments. Consistent with previous reports [26, 27], TGF-β1 induced significant mitochondrial ROS production in a dose-dependent manner compared to control cells (Fig. 3a). Both pre- and post-treatment of cells with amifostine significantly reduced mitochondrial ROS levels (Fig. 3b). Mitochondrial membrane potential and intracellular ATP levels were also measured to assess the effects of amifostine on mitochondrial function. TGF-β1 significantly reduced the mitochondrial potential compared to the control, as reflected by the ratio of green to red fluorescence in JC-1 staining. In contrast, post-treatment with amifostine significantly restored the loss of mitochondrial membrane potential induced by TGF-β1 (Fig. 3c). Similarly, intracellular ATP levels were downregulated by TGF-β1 in a dose-dependent manner (Fig. 3d), while amifostine treatment rescued the ATP level in a dose-dependent manner (Fig. 3e).

### Amifostine attenuates the reprogramming of TGF-β1-induced metabolism in fibroblasts

**Fig. 4:**
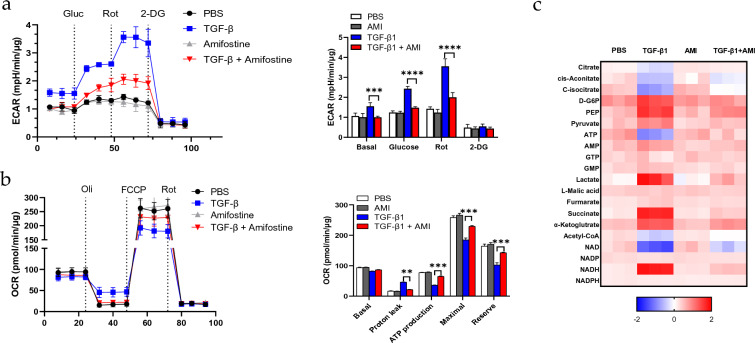
Amifostine restores energy metabolism homeostasis.** a** The extracellular acidification rate was measured as a reflection of cell metabolism. Cells were pretreated with TGF-β1 for 30 min followed by treatment with amifostine for another 30 min before the assay. Glucose (Gluc), rotenone (Rot) and 2-DG were sequentially added into the medium at 20 min intervals. The medium pH value was monitored and normalized to the cell lysate protein contents. *n* = 5, ***p < 0.001, ****p < 0.0001. **b** The oxygen consumption rate was measured by sequential addition of oligomycin (Oli), carbonyl cyanide-4 (trifluromethoxy) phenylhydrazone (FCCP), and rotenone + antimycin A (Rot). *n* = 5, **p < 0.01, ***p < 0.001. **c** Metabolites were analyzed by MS. n = 3, Data are presented as a fold-change compared to the basal control level of individual metabolites

Mitochondrial dysfunction is closely related to altered cell metabolism, which is increasingly recognized as a hallmark of fibrosis [6]. We examined whether TGF-β1 affects the cellular metabolic profile by measuring the extracellular acidification rate (ECAR) and the oxygen consumption rate (OCR). As shown in Fig. 4a, TGF-β1 increased the basal ECAR compared to the saline control, suggesting that it enhances glycolysis. Amifostine by itself had no effect on ECAR, but it mitigated the increase in basal ECAR caused by TGF-β1 (Fig. 4a). This was further confirmed by the following observations. When glucose was added to the medium, TGF-β1-treated cells showed a dramatic increase in ECAR, suggesting the added glucose was promptly used for glycolysis. Although the ECAR in amifostine-treated cells was elevated after glucose addition, the magnitude was greatly attenuated. When respiratory chain complex I was inhibited by rotenone, the tricarboxylic acid (TCA) cycle was blocked and the cells showed obvious glycolysis turnover. This was especially significant in TGF-β1-treated cells. In contrast, amifostine markedly reversed this shift. Furthermore, inhibition of hexokinase by 2-deoxy-D-glucose (2-DG) completely blocked glycolysis and TCA cycle- relevant acidification in all groups.

We next examined the effects of TGF-β1 on the OCR, which reflects the ability of cells to consume oxygen and to generate ATP. After blocking ATP synthase activity with oligomycin treatment, TGF-β1-treated cells showed less reduction in OCR compared to other groups (Fig. 4b). This implies that ATP generation in TGF-β1-treated cells is more likely to occur through the process of glycolysis. It also implies that TGF-β1 caused more proton leaks from the space between the outer and inner mitochondrial membranes. Carbonyl cyanide-4 phenylhydrazone (FCCP) collapses the proton gradient and disrupts the mitochondria membrane potential, thereby inducing maximal ATP generation through proton gradient collapse. TGF-β1-treated cells showed less ATP generation compared with control cells. In contrast, treatment with amifostine rescued the decline in ATP generation caused by TGF-β1.

We next investigated whether amifostine can modulate metabolic reprogramming induced by TGF-β1 in vitro by examining 20 metabolites involved in glycolysis and in the mitochondrial TCA cycle. TGF-β1 treatment markedly altered the metabolic profile and shifted metabolism towards glycolysis (Fig. 4c and Table 1). The concentrations of glycolysis metabolites (glucose-6-phosphate, phosphoenolpyruvate, pyruvate, lactate) were significantly increased in response to TGF-β1. In contrast, the concentrations of TCA cycle metabolites (acetyl-CoA, citrate, cis-aconitate, c-isocitrate) were decreased. However, succinate was upregulated by TGF-β1. Consistent with these observations, the ATP level was downregulated by TGF-β1, while the AMP levels were upregulated. The most altered metabolites were NAD and NADH. Importantly, amifostine restored these metabolites to normal levels. The above results indicate that fibroblasts adapt to glycolysis under fibrotic conditions, and that amifostine corrects the altered metabolic profile associated with a fibrotic phenotype.

**Table 1:** Metabolites change after TFG-β1 challenge

Metabolites	Mean Diff (PBS vs TGF-β1)	Adjusted *P* value
Citrate	0.37	< 0.0001
cis-Aconitate	0.7733	< 0.0001
C-isocitrate	1.207	< 0.0001
D-G6P	− 0.56	< 0.0001
PEP	− 1.027	< 0.0001
Pyruvate	− 0.52	< 0.0001
ATP	1.543	< 0.0001
AMP	− 0.3067	0.0005
GTP	0.04	0.9532
GMP	− 0.1767	0.0992
Lactate	− 1.543	< 0.0001
L-Malic acid	− 0.05	0.9137
Furmarate	− 0.19	0.0656
Succinate	− 1.543	< 0.0001
α-Ketoglutrate	− 0.82	< 0.0001
Acetyl-CoA	0.65	< 0.0001
NAD	− 1.62	< 0.0001
NADP	0.03333	0.9721
NADH	1.71	< 0.0001
NADPH	0.01333	0.9981

### AMPK mediates the anti-fibrotic effects of amifostine by stabilizing mitochondrial homeostasis

**Fig. 5:**
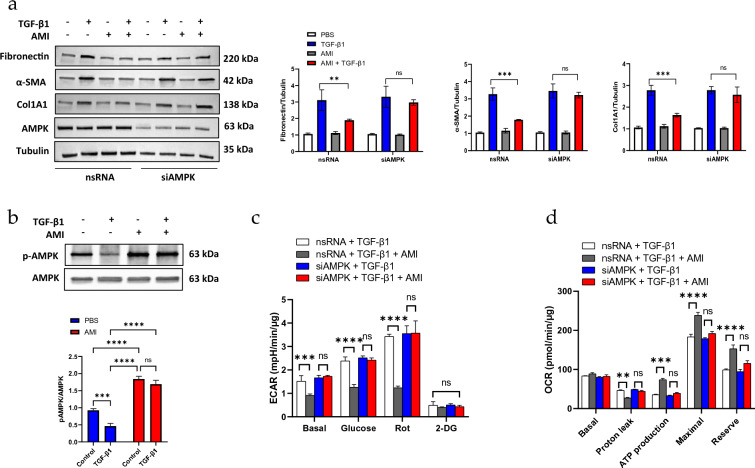
AMPK mediates the anti-fibrosis effects of amifostine.** a** Human fibroblast cells were transfected with nsRNA or siAMPK for 48 h prior to TGF-β1 and/or amifostine treatment. Fibronectin, α-SMA and Col1A1 levels were quantified by Western blot. Knockdown of AMPK was also confirmed by Western blot. *n* = 3, **p < 0.01, ***p < 0.001. The results shown are cropped from the original blots. **b** The effects of TGF-β1 and amifostine on AMPK activation were detected with the Thr172 phosphorylated AMPK antibody. The results shown are cropped from original blots. **c** Fibroblast cells were transfected with nsRNA or siAMPK for 48 h. The extracellular acidification rate was then measured as a reflection of cell metabolism. Cells were pretreated with TGF-β1 for 30 min, followed by amifostine treatment for another 30 min before the assay. Glucose (Gluc), rotenone (Rot) and 2-DG were added sequentially into the medium at 20 min intervals. The medium pH value was monitored and normalized to the cell lysate protein contents. n = 5, ***p < 0.001, ****p < 0.0001. **d** The oxygen consumption rate was measured by sequential addition of oligomycin (Oli), carbonyl cyanide-4 (trifluromethoxy) phenylhydrazone (FCCP) and rotenone + antimycin A (Rot). Data was normalized to the cell lysate protein contents. *n* = 5, **p < 0.01, ***p < 0.001

Loss of AMPK activity may contribute to the mitochondrial dysfunction and metabolic reprogramming that characterizes the profibrotic phenotype of fibroblasts [28]. We hypothesized that AMKP could mediate the anti-fibrotic effects of amifostine. siRNA-mediated gene knockdown of AMPK in fibroblasts resulted in comparable collagen, fibronectin, and α-SMA expression levels to those induced by TGF-β, even in the presence of amifostine (Fig. 5a). This suggests the anti-fibrotic effects of amifostine are dependent on AMPK activity. To further evaluate the role of AMPK, the effects of TGF-β1 and amifostine on AMPK phosphorylation were examined. TGF-β1 significantly reduced AMPK phosphorylation, while amifostine blocked TGF-β1 downregulation of AMPK phosphorylation (Fig. 5b). Interestingly, amifostine by itself also induced significant AMPK phosphorylation. Next, we further confirmed the role of AMPK in mediating the effects of amifostine on mitochondrial metabolism. Like Fig. 4a, TGF-β1 expediated extracellular acidification rate when glucose or rotenone was added into nsRNA-transfected cells, and this effect was inhibited by amifostine. In contrast, in siAMPK RNA-transfected cells, the inhibitory effect of amifostine was significantly diminished (Fig. 5c). The AMPK-dependent effects of amifostine were further demonstrated with OCR measurement. Compared with nsRNA-transfected cells, siAMP RNA-transfected cells showed similar glycolysis-dependent ATP generation in TGF-β1-treated cells no matter the presence of amifostine (Fig. 5d).

### Amifostine induces AMPK activation in a SIRT1-dependent manner

**Fig. 6:**
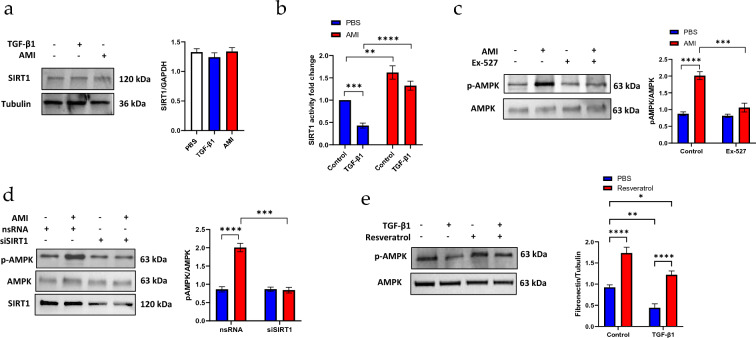
Amifostine-induced AMPK activation is mediated by SIRT1. **a** Western blot detection of SIRT1 expression in human fibroblast cells stimulated by TGF-β1 or amifostine. *n* = 3. The results shown are cropped from the original blots.** b** SIRT1 activity assay was used to determine SIRT1 activity in human fibroblast cells after stimulation by TGF-β1 or amifostine treatment. SIRT1 activity is plotted as a fold-change compared to PBS control cells. *n* = 5, ***p < 0.001.** c** SIRT1 was inhibited by its specific inhibitor Ex-527 (5 µM, 30 min). Cells were then treated with amifostine. Activation of AMPK was detected with the Thr172 phosphorylated AMPK antibody. The results shown are cropped from the original blots. **d** Endogenous SIRT1 was downregulated by transfection with SIRT1 siRNA. Phosphorylation of AMPK at Thr172 was detected by Western blot. *n* = 3, *p < 0.05, ***p < 0.001, **** < 0.0001. The results shown are cropped from original blots. **e** Cells were treated with SIRT1 activator resveratrol (10 µM, 30 min) followed by TGF-β1 treatment. Phosphorylation of AMPK was determined by Western blot. *n* = 3, *p < 0.05, **p < 0.01, ***p < 0.001, **** < 0.0001

**Fig. 7:**
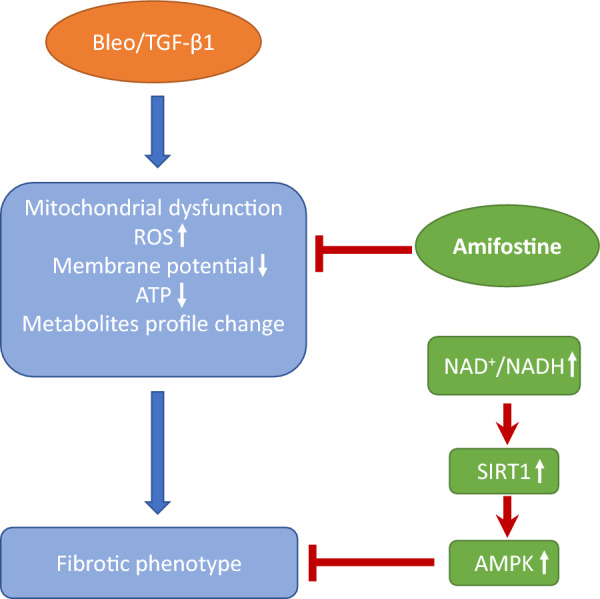
Schematic mechanism for the therapeutic effects of amifostine on IPF. The Profibrotic stimuli bleomycin and TGF-β1 induce comprehensive mitochondrial dysfunction due to increased ROS production, loss of mitochondrial membrane potential, and changes in the metabolite profile. Amifostine inhibits excessive mitochondrial ROS production, restores the mitochondrial membrane potential, and maintains homeostasis of mitochondrial metabolism. Restored NAD^+^ activates SIRT1 and its downstream kinase AMPK, which in turn inhibits the development of pulmonary fibrosis

Next, we explored the molecular mechanism of amifostine-induced AMPK activation. Amifostine is known to upregulate the level of NAD, which functions as a cofactor for multiple cellular enzymes. SIRT1 is a NAD-dependent deacetylase that regulates gene expression and cellular metabolic status. We therefore investigated whether amifostine-induced AMPK activation is mediated by SIRT1. Both amifostine and TGF-β1 did not alter the SIRT1 expression level (Fig. 6a). However, TGF-β1 markedly inhibited SIRT1 activity (Fig. 6b). In contrast, amifostine by itself significantly increased SIRT1 activity and also counteracted the TGF-β1 effect on SIRT1 activity. We also investigated if TGF-β1 alters the intracellular localization. As shown in the Additional file 1: Figure S1, SIRT1 was largely localized in the nucleus, TGF-β1 treatment didn’t cause any change of SIRT1 localization. To study the link between SIRT1 and AMPK, cells were treated with SIRT1 inhibitor, Ex-527, or transfected with SIRT1 siRNA. Amifostine induced significant phosphorylation of AMPK, which was inhibited by Ex-527 (Fig. 6c). Nonetheless, amifostine induced marked phosphorylation of AMPK in nsRNA-transfected cell, while the phosphorylation of AMPK by amifostine was significantly blocked in siSIRT1-transfected cells (Fig. 6d). To further confirm the activation of AMPK is SIRT1 dependent, cells were pre-treated with SIRT1 activator, resveratrol, at 10 µM for 30 min followed by TGF-β1 stimulation. TGF-β1 significantly reduced AMPK phosphorylation compared with control cells. Resveratrol significantly blocked downregulation of AMPK phosphorylation by TGF-β1. Interestingly, resveratrol markedly upregulated AMPK phosphorylation. These results clearly indicate that AMPK activation by amifostine was mediated by SIRT1 (Fig. 7).

## Discussion

IPF is characterized by the formation of scar tissue composed of extracellular matrix (ECM). This causes thickening loss of tissue mobility and impaired organ function. The mechanisms involved in fibrosis are fundamentally similar to those that occur in the normal wound healing process. Fibroblasts play a pivotal role in excess tissue synthesis and deposition, as well as in ECM remodeling [29]. There is a growing appreciation that core biosynthetic and bioenergetic metabolic pathways are perturbed in fibrosis disease [30]. In particular, mitochondrial dysfunction and metabolic reprogramming have been linked to the pathogenesis of IPF via a range of fundamental biological processes including mitochondrial fusion–fission imbalance, mitophagy dysfunction, cell senescence, etc.

In the present study, TGF-β1 was found to markedly alter fibroblast energy metabolites in favor of glycolysis, as manifested by the accumulation of D-G6P, PEP and lactate. In contrast, the TCA metabolites of citrate, cis-aconitate, c-isocitrate and acetyl-coA were significantly reduced by TGF-β1. Moreover, the ratios of AMP/ATP and of NAD^+^/NADH were downregulated by TGF-β1, suggesting the fibrotic phenotype has a lower energy consumption. Importantly, amifostine treatment restored the altered metabolite status and mitochondrial function. The most rescued metabolites by amifostine treatment were NAD^+^ and NADH, both of which are essential cofactors for numbers of cellular processes. NADH is mainly generated by TCA. Although glycolysis also generates NADH, this is converted from pyruvate to lactase, and the net gain of NADH from glycolysis is therefore zero. The initial step in the mitochondrial respiratory chain is the oxidation of NADH by complex I. In this step, electrons are removed from NADH and transferred to co-enzyme Q, while NADH is converted back to NAD^+^ and H^+^. Consequently, the ratio of NAD^+^ to NADH infers the state of equilibrium between OXPHOS and glycolysis. Higher NAD^+^ suggests a shift towards OXPHOS, and vice versa. In the present study, TGF-β1 significantly decreased NAD^+^ and increased NADH. This may be due to impaired mitochondrial respiratory function which blocks NADH utilization, resulting in accumulation of NADH and less conversion of NADH into NAD^+^. The ratio of NAD^+^ to NADH was also observed to decrease. Collectively, these results show that TGF-β1 clearly impairs mitochondrial function. Amifostine is a ROS scavenger and both pretreatment and post-treatment of fibroblast with this drug significantly reduced the level of mitochondrial ROS. The reduction in mitochondrial oxidative stress also improved mitochondrial function. Consequently, OXPHOS could be refueled by NADH and the ratio of NAD^+^ to NADH returned to normal levels.

In addition to fueling ATP production via OXPHOS, NAD + is critical for several vital functions and can thereby influence numerous pathways related to disease pathogenesis [31]. Importantly, NAD^+^ is the rate-limiting cofactor for SIRT1, which has been shown to exert regulatory control over glycolysis [32, 33] and OXPHOS [34, 35]. The present study found that amifostine did not induce SIRT1 expression, but instead increased SIRT1 activity. This can be attributed to upregulation of NAD^+^ by amifostine. Our data also indicate that TGF-β1 downregulated SIRT1 activity but didn’t induce its production and change in localization. The potential mechanism that TGF-β1 inhibits SIRT1 activity may through ROS induced by TGF-β. TGF-β has been widely reported to induce intracellular ROS in various cells. TGF-β1 can induce ROS production through several mechanisms including induction of NADPH oxidase 4 expression, increasing mitochondrial ROS, which was evidenced in the current study, and inhibition of antioxidants [36–38]. Meanwhile, SIRT1 activity can be inhibited by oxidation of its cysteine residue and by ROS-dependent intracellular NAD + depletion [39, 40]. Based on the above evidence, it’s possible that TGF-β1 inhibits SIRT1 activity through increased oxidative stress and subsequent depletion of NAD + , which was demonstrated in this study. SIRT1 is a well-known regulator of fuel-sensing [41]. Similarly, AMPK is also an important regulator of energy metabolism [28, 42], and its activity is decreased in humans with IPF and in an experimental mouse model of lung fibrosis [43]. Pharmacological activation of AMPK in myofibroblasts from human lungs with IPF display lower fibrotic activity [43]. Both SIRT1 and AMPK play critical role in cellular fuel metabolism. They regulate each other and share many common target molecules. AMPK is activated by reversible phosphorylation of the threonine residue 172 on its catalytic α unit. This is carried out by two kinases: serine-threonine liver kinase B1 (LKB1) and calcium/calmodulin kinase kinase β (CaMKKβ). There is some evidence linking SIRT1 and AMPK activation. SIRT1 can diminish lysine acetylation of LKB1 and cause it to translocate from the nucleus to the cytoplasm. Here, LIKB1 associates with the adaptor proteins STE20-related adaptor protein and mouse embryo scaffold protein, resulting in the activation of AMPK [44]. Furthermore, increased lysine acetylation of LKB1 correlates with decreased LKB1 and AMPK activity. Other evidence has shown that activation of AMPK by polyphenols depends on the presence of both SIRT1 and LKB1 [45]. In addition, the SIRT1 inhibitor NAM downregulated the activity of both AMPK and SIRT1 [46]. The current results showed that amifostine-induced AMPK activation was dependent on SIRT1 activation. LKB1 may, therefore, link the activation of SIRT1 and AMPK, although further investigations are needed to confirm the effect of amifostine on LKB1 acetylation levels.

In addition to changes in NAD^+^/NADH, other metabolites were also altered by TGF-β1. Lactate is a marker of anaerobic respiration and is converted from pyruvate by lactate dehydrogenase (LDH). Lactate accumulation results from dysregulated glycolysis. Increased lactate levels have been reported in the lung tissue of patients with IPF [47]. Our results indicate that TGF-β1 upregulates lactate production, but this can be inhibited by amifostine. These findings further highlight the effects of amifostine on metabolism homeostasis. Succinate is another metabolite that increased in response to TGF-β1 but decreased after amifostine treatment. Succinate is known to be a signaling molecule and can bind to its specific receptor, GPR91, to enhance the inflammatory response [48]. GPR91 expression has been correlated with the degree of fibrosis in the liver [49], while α-SMA-positive fibroblasts colocalize with GPR91 [50]. Downregulation of succinate by amifostine could, therefore, be a consequence of improved mitochondrial function.

Other mechanisms could also exist and warrant further investigation. One potential mechanism is that amifostine protects mitochondrial DNA (mtDNA). Amifostine is known to protect cells against cytotoxic DNA damage induced by ionizing radiation or DNA-damaging chemotherapeutic agents [20, 51]. Once amifostine enters cells it can be converted into the disulfide WR-33278, which shares structural similarity with polyamines [52]. Both the thiol format and the disulfide derivative of amifostine can remove platinum adducts from DNA. mtDNA is more prone to damage than nuclear DNA because it is not protected by histone proteins. mtDNA damage and the impaired repair of this damage has been associated with IPF [15, 53, 54]. Hence, it is plausible that amifostine exerts its effect on mitochondrial function by protecting mtDNA from damage. Although the present study did not explore this possibility, it will be interesting in future studies to investigate the mechanism by which amifostine protects against mtDNA damage. This study is limited in its ability to elucidate the impact of amifostine on lung parenchymal morphology and comprehensive profiling of anti-inflammatory cytokines. Further research is warranted to delve into these two aspects.

In summary, as illustrated in Fig. 7, amifostine restores mitochondrial function and homeostasis via multiple mechanisms. The key anti-fibrotic effect of amifostine is its ability to correct the NAD^+^/NADH ratio, which in turn activates SIRT1 and subsequently AMPK. These pathways ultimately lead to metabolic reprogramming. Amifostine is already a clinically used drug and could, therefore, be repurposed as a novel regime for IPF therapy.

### Supplementary Information

**Additional file 1: Supplementary Figure S1.** Human fibroblast cells were treated with PBS or TGF-β1 for 2 hours. Cells were washed with PBS for 2 times and fixed with 3.7% formaldehyde at room temperature for 10 min and permeabilized with 0.1% Triton X-100. Cells were incubated with SIRT1 antibody (1:100 in 2% BSA/PBS) at room temperature for 1 h followed by Alex Fluor-568 secondary antibody incubation. Cell nucleus was stained with DAPI in mounting media. Cells were visualized with 40x confocal microscope. SIRT1 was observed primarily in the nucleus in both PBS- and TGF-β1-treated cells.

## Data Availability

Not applicable.
