# Supplementary material for: Amifostine ameliorates bleomycin-induced murine pulmonary fibrosis via NAD+/SIRT1/AMPK pathway-mediated effects on mitochondrial function and cellular metabolism
Source: Eur J Med Res. 2024 Jan 20;29:68. doi: 10.1186/s40001-023-01623-4 (PMC10799491; doi:10.1186/s40001-023-01623-4)
Supplement: Supplementary file 1 — Additional file 1: Supplementary Figure S1. Human fibroblast cells were treated with PBS or TGF-β1 for 2 hours. Cells were washed with PBS for 2 times and fixed with 3.7% formaldehyde at room temperature for 10 min and permeabilized with 0.1% Triton X-100. Cells were incubated with SIRT1 antibody (1:100 in 2% BSA/PBS) at room temperature for 1 h followed by Alex Fluor-568 secondary antibody incubation. Cell nucleus was stained with DAPI in mounting media. Cells were visualized with 40x confocal microscope. SIRT1 was observed primarily in the nucleus in both PBS- and TGF-β1-treated cells. [file 40001_2023_1623_MOESM1_ESM.pdf]

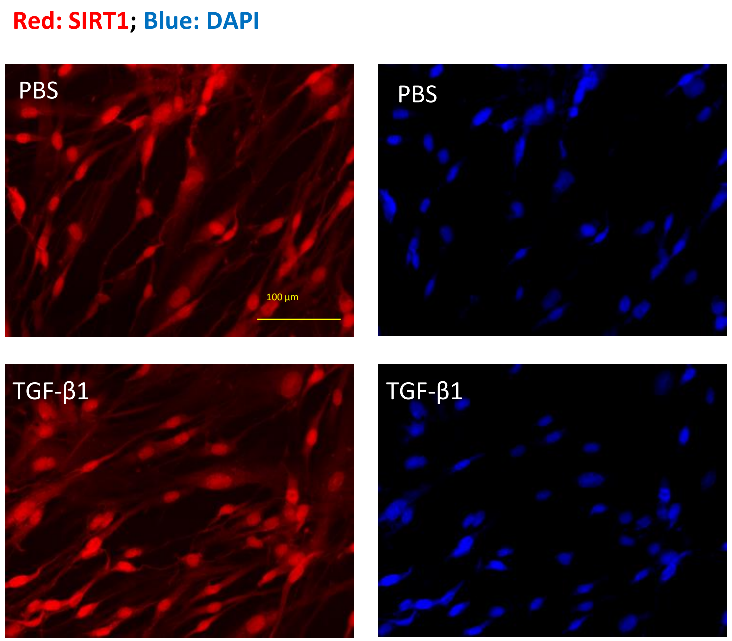

Supplementary Figure S1

Human fibroblast cells were treated with PBS or TGF-β1 for 2 hours. Cells were washed with PBS for 2 times and fixed with 3.7% formaldehyde at room temperature for 10 min and permeabilized with 0.1% Triton X-100. Cells were incubated with SIRT1 antibody (1:100 in 2% BSA/PBS) at room temperature for 1 h followed by Alex Fluor-568 secondary antibody incubation. Cell nucleus was stained with DAPI in mounting media. Cells were visualized with 40x confocal microscope. SIRT1 was observed primarily in the nucleus in both PBS- and TGF-β1-treated cells.
